# Rationale and design of randomized controlled trial protocol of cardiovascular rehabilitation based on the use of telemedicine technology in the Czech Republic (CR-GPS)

**DOI:** 10.1097/MD.0000000000012385

**Published:** 2018-09-14

**Authors:** Ladislav Batalik, Filip Dosbaba, Martin Hartman, Katerina Batalikova, Jindrich Spinar

**Affiliations:** a Department of Rehabilitation, University Hospital Brno; b Department of Internal Cardiology Medicine— Institutions Shared with the Faculty Hospital Brno—Adult Age Medicine—Faculty of Medicine Brno, Brno, Czech Republic.

**Keywords:** cardiac rehabilitation, cardiovascular disease, Czech Republic, physical fitness, quality of life, telemedicine

## Abstract

**Background:**

Cardiovascular diseases remain the most common causes of death in the world. Instructions for secondary prevention recommend multifaceted approach in cardiovascular diseases risk management. Center-based physical exercise training is considered as an important integral part of cardiac rehabilitation (CR). Despite all recognized benefits CR brings, active interest of patients remains low in many countries, including the Czech Republic. That is why there is a need to focus on more effective patients’ participation in CR with respect to their preferences and needs. One of possible approaches is using telemonitoring guidance based on obtaining data via technological equipment during home exercise training. The aim of this study is to compare effectiveness of both center- and home-based exercise training with focus on participants’ physical fitness and quality of life.

**Methods/design:**

This randomized control trial intends to monitor cardiorespiratory health indicators and quality of life of patients diagnosed with a coronary artery disease (CAD) at the University Hospital Brno, Czech Republic. These patients will be randomly separated into 2 groups—a regular outpatient group (ROT) and an intervention training group (ITG). Both groups undergo a 12-week rehabilitation training program. The ROT group will undergo center-based exercise trainings in the hospital and receive feedback and support directly by their coach. The ITG group will be telemonitored during exercise training in their home environment via a wrist sport tester and Internet application.

All patients will be supposed to exercise at 70% to 80% of their heart rate reserve obtained from cardiopulmonary exercise test (CPX). The primary outcome is to measure and compare physical fitness values assessed at baseline and after 12 weeks of training. Physical fitness is expressed as peak oxygen uptake assessed by the CPX test. The secondary outcomes are patients, training adherence, and their quality of life.

**Discussion:**

This trial focuses on an up-to-date topic. As there have not been any similar trials in the Czech Republic yet, we expect it to bring great benefits not only for our hospital in Brno. In the long term, this method seems to be low-cost for all participants and brings a lot of benefits for those patients, who are for many reasons unable to participate in center-based CR provided by hospitals and other health care centers. Physical exercise therapy brings good results in reducing cardiovascular risk factors and improves its global impact. Thanks to its simplicity, it is expected to increase patients’ training adherence as well.

## 1 Background

Cardiovascular diseases have been, already for a long time, the most common causes of death in the world.^[1]^ These diseases are related to high blood pressure, cholesterol, diabetes, and smoking.^[2]^ The most common cardiovascular causes of death are coronary artery diseases (CAD) and cerebrovascular diseases. In 2014, the CAD caused 126 deaths per 100,000 inhabitants in the whole EU-28. The standardized CAD mortality rate in the Czech Republic in 2014 was 333.1 deaths per 100,000 inhabitants. One year later the Czech Statistical Office published its results, proving that 42% of men and 50% of women deaths were caused by CAD. On the whole, CAD caused 51% death cases from the whole number of 111,173 deaths, which is alarming 46%.^[3]^

Health care in the Czech Republic system is being financed mainly from public health insurance, some patients partially cover the costs themselves just in occasional cases. In an international comparison of health care expenses, the Czech Republic is highly above the average in Europe (42%).^[4]^

Czech health insurance companies pay most money for cardiovascular diseases treatment. In 2016 it was 25.4 billion CZK, which was 10.7% of all the health care expenses that year.^[5]^

With life expectancy still increasing (between 2006 and 2016 life expectancy grew from 73.4 to 79.7 for men and from 76.2 to 81.2 for women)^[6]^ and medical treatment improving, these expenses are expected to grow significantly in the future.^[7]^ It is necessary to deal with this fact and search for solutions how to prevent or at least significantly reduce these diseases.

One of possible ways of cardiovascular diseases prevention is a cardiac rehabilitation as a complex secondary intervention, where physical exercise training plays crucial role.

At the same time, it shows people how to develop, accept, and lead a healthy lifestyle.^[8]^

Heran et al's study discovered that CR can reduce the total mortality rate by 13% and cardiovascular mortality by 26%.^[9]^ The Czech Republic provides this preventative care only in hospitals and medical centers under a specialist's supervision.^[10]^ Despite recognized benefits, the active patients’ participation remains low.^[11]^

Main factors why patients miss the CR training seem to be technical complications. Above all, they mention commuting difficulties, time demandingness, and concrete training program timing. A social factor plays an important role too.^[12]^

Another disadvantage of CR guided by hospitals is its momentariness. Patients undergo a prescribed monitored training program and then they are discharged from the hospital without any possibility to get feedback regarding blood pressure, heart rate, or individual training care.^[13]^ That is why it is necessary to focus on the possibility of a long-term CR intervention besides the one provided by hospitals and medical centers.

One of the effective ways is to move a part of CR to patient's home environment. Meta-analysis^[14]^ of the Cochrane health database revealed insufficient differences between home- and hospital-based CR training from a short-term point of view. However, studies evaluating long-time effects referred to more significant results. For example, Smith et al's study ^[15]^ showed a decline in peak VO_2_ between 1 and 6 years follow-up in both the home- and hospital-based group, but the rate was significantly smaller in the home-based group.

Committee for Practice Guidelines of the European Society of Cardiology^[16]^ came to conclusion that CR should focus not only on risk factors modification and patients’ pharmacological adherence, but it should also provide patients with other different possibilities, such as using telemedicine.

Systematic review of earlier telemedical studies revealed a significant influence of cardiovascular risk factors, including adherence, total cholesterol, HDL cholesterol, and systolic blood pressure.^[17]^

Although the current findings highlighted benefits of telemedicine, previous researches confined themselves to technologies binding patients to concrete places. It is necessary to explore technologies supporting flexibility of training program.

Web and mobile technologies are receiving attention as an alternative approach to support changes in human behavior, clinical improvement, and better social function of patients.^[18]^ For example, providing care via short message system can help in curing many chronic diseases, such as diabetes and asthma.^[19]^ Recently, finished randomized study Telerehab III^[20]^ presented an intervention using short message system and Internet in mobile phone as an efficient, cost-effective way to provide health care. In comparison to a control center-based group of patients, who got a usual care, patients in home-based group using mobile phones pointed out that their quality of life and motivation to be physically active have significantly increased.

This study was also an important source of information about how mobile phones can be effective in modern medicine. However, it is necessary to monitor physical exercise trainings in detail to provide patient with information about required heart rate, intensity, and training duration.^[21]^ Practicability of distant monitoring has been recently proved by using smartphones, ECG sensors, and GPS device.^[22]^ The 6-week-long program decreased patients’ depression and improved walking performance and physical health related to quality of life.

Fit@Home study^[23]^ from 2017 presented positive results when using a heart rate monitor. However, it mentions great limitations, such as lack of blinding for the physician regarding patient's allocation, and some patients’ discomfort while wearing the chest strap of the heart monitor.

Nevertheless, telemedicine brings great benefits, although it needs another randomized control studies to get more statistically significant data related to this approach.

## 2 Methods

Participants of CR-GPS project, as a single prospective randomized controlled trial, will be the patients of University Hospital (UH) Brno, Czech Republic, above the age of 18, diagnosed with CAD (angina pectoris, myocardial infarction, patients after coronary revascularization) in the last 6 months, with left ventricular ejection fraction >45%.

All eligible patients will be recommended by physiotherapists and research assistants from UH Brno. They must be clinically stable, able to undergo CPX, understand and write in Czech, and agree to participate in this project. Due to a random separation into 2 groups, all of them must have a mobile phone and free access to Internet connection.

Patients participating in CR-GPS will be further classified by cardiologist according to following criteria:

- No significant cardiovascular risk
- No implanted cardioverter-defibrillator or pacemaker
- No residual partial coronary artery stenosis requiring revascularization
- No orthopedic or neurological disability to exercise
- No mental disadvantage making cooperation impossible
- No serious oncological disease or treatment

During CR-GPS, some health problems might occur. If physical status of any participant changes significantly, he or she will be examined by the cardiologist who might change the medication. If this health problem becomes serious, this patient will be excluded from the trial. The obtained data of such patient will be further secured but not involved in final statistical evaluation.

According to Vysoký et al's study,^[24]^ number of participants calculation will be based on the improvement of peak VO_2_ 3.2 mL/kg/min with standard deviation 4.2 mL/kg/min. We will need 56 participants to reach 80% of statistical power with significance level set at *P* < .05. We count with 10% participant loss during the trial.^[25]^

At the beginning of the trial, all participants undergo baseline assessment on cycle ergometer (Ergoline Ergoselect 100, Bitz, Germany) and cardiologist will test their physical fitness through CPX. After that, they obtain a study package containing personal data questionnaire (sex, age, weight, diagnosis, pharmacological treatment), list of trial information, and informed consent form.

Personal information will be sent to research team from UH Brno. They will be processed and backed up according to currently valid General Data Protection Regulations (GDPR). A total of 56 patients will be separated to ITG using a telemedicine assistance and ROT group with regular center-based CR. This separation will be random, done by a computerized allocation system applying an algorithm in proportion 1:1. Investigators will not be aware of the randomized matching sequence. The patient and care providers are not blinded to the intervention.

### 2.1 Training description

#### 2.1.1 Intervention training group

Each ITG patient will be lent a wrist sport tester (Polar M430, Kempele, Finland) which monitors heart rate and other training values—time, training mode, duration, and distance.

A training session in ITG group consists of the following:

1. Part—warm up—10 minutes
2. Part—aerobic phase—60 minutes walking or cycling (according to predefined training heart rate set at 70% to 80% heart rate reserve)
3. Part—cool down—10 minutes

Training period is set to 60 minutes per one session 3 times a week for 12 weeks.

To know what to do and how to train at home, the first 2 training sessions will be controlled by a physiotherapist at the CR clinic in the hospital. Further training will take place in home conditions. Training data (heart rate a training values) will be recorded by the wrist sport tester. Each patient will be supposed to download the data to a Polar Flow web application (secured by login and password). Each patient will get his or her own login and password. Physiotherapists will have access to all patients’ accounts to be able to check the data. These data will be further backed up on a separate secured external hard drive, and later processed and evaluated by the trial investigator.

The great advantage of using this web application is a possibility to use it as a diary. Each patient and physiotherapist can view training history; they can follow the results to see whether they are improving or not. Physiotherapists have the role of coaches who check their patients via telephone once a week and give them feedback including recommendations, advice, and training motivation.

#### 2.1.2 Regular outpatient training group

Patients in ROT group undergo a physical exercise workout period under the direct supervision of a physiotherapist specializing in CR. The patients will exercise on cycle ergometers (GE E-Bike Basic, Boston, MA) and treadmills (Forcelink BV, Amsterdam, The Netherlands).

A training session in ROT group consists of the following:

1. Part—warm up—10 minutes
2. Part—aerobic phase—60 minutes cycling on ergometers and walking on treadmill (according to predefined training heart rate set at 70%–80% heart rate reserve)
3. Part—cool down—10 minutes

Training period is set to 60 minutes per one session 3 times a week for 12 weeks.

A physiotherapist will monitor each patient's heart rate and blood pressure. Measured data will be recorded on the secured external hard drive. At the end of study (after 12 weeks of training) all participants will be tested again. The main variable monitored is physical fitness assessed by CPX and SF-36 questionnaire. Physiotherapist will monitor the whole study process, and give patients of both groups proper feedback with possibility to consult any problems and difficulties related to training, technical issues, and their physical condition.

#### 2.1.3 Outcomes

**Figure 1:**
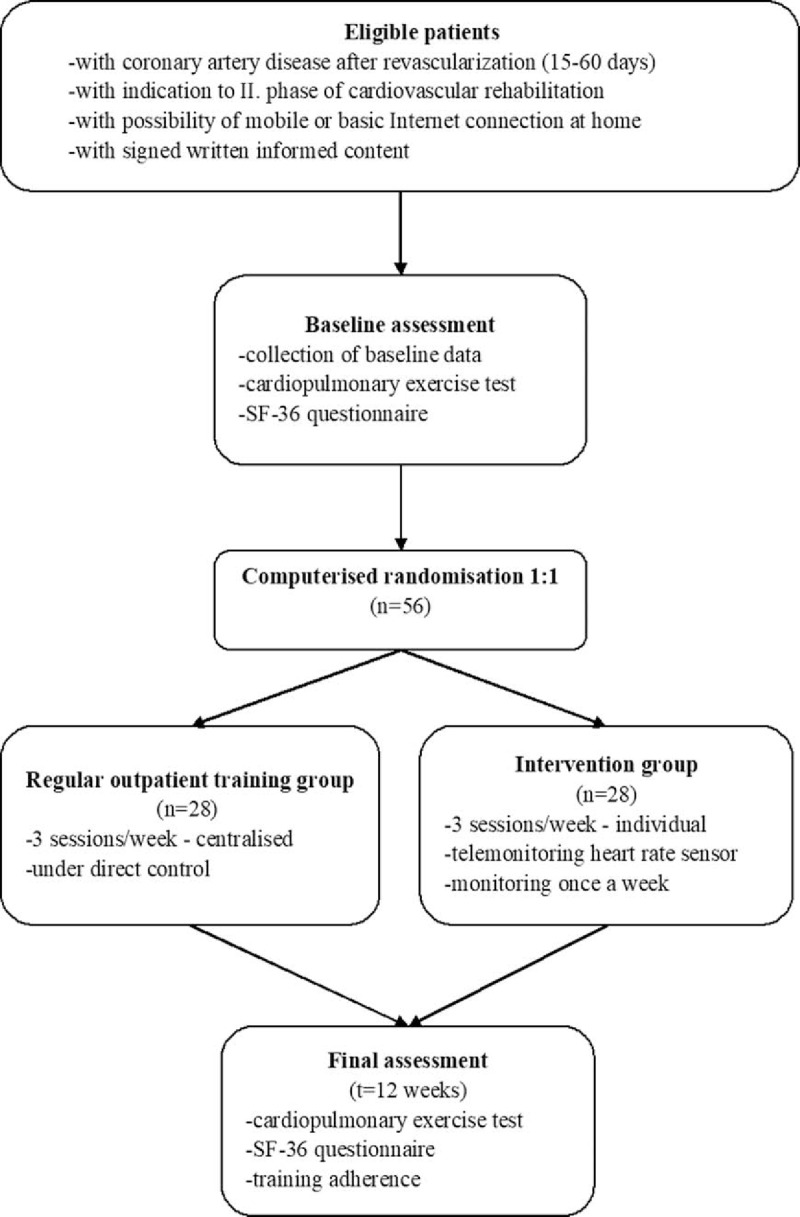
Flowchart of the study protocol.

Main outcome is to compare physical fitness values measured at baseline and after 12 weeks in 2 groups of patients (ROT and ITG). Secondary outcomes are health related quality of life and training adherence. Health-related quality of life is measured at baseline and after 12 weeks. Training adherence is assessed in both groups during the 12 weeks of exercise period. A summary of the trial design is provided in flowchart (Fig. 1).

## 3 Measurements

### 3.1 Physical fitness

Physical fitness will be measured by individual maximal CPX test with respiratory gas analysis performed on bicycle ergometer (Ergoline Ergoselect 100) using a ramp protocol. This test consists of 8 to 12 minutes cycling at 60 to 70 rpm frequency. A 12 lead ECG and blood pressure are recorded continuously during this test. Peak VO_2_ will be determined as the mean value of the last 30 seconds of exercise. Patients will be encouraged to exercise until they reach respiratory exchange ratio ≥1.10. Oxygen consumption will be measured by Metalyzer 3b (Cortex Biophysics GmbH, Leipzig, Germany).

### 3.2 Health quality of life

Data related to patients’ quality of life will be assessed by an SF-36 questionnaire. The SF-36 consists of 8 scaled scores, which are the weighted sums of the questions in their section. Each scale is directly transformed into a 0 to 100 scale on the assumption that each question carries equal weight.

The 8 sections are as follows:

1. Vitality
2. Physical functioning
3. Bodily pain
4. General health perceptions
5. Physical role functioning
6. Emotional role functioning
7. Social role functioning
8. Mental health

Each patient will fill this questionnaire at baseline and after 12 weeks of training. These data will be statistically processed.

### 3.3 Training adherence

Training adherence is defined as a total number of accomplished training sessions. Date will be assessed via Polar flow web application in ITG and a session attendance checklist in ROT group.

### 3.4 Statistical analysis

Baseline characteristics assessed from the questionnaire will be summarized using descriptive statistics. A paired *t* test will be used to find out whether there are statistically significant changes between baseline and final physical fitness values and values describing health-related quality of life. Between-group differences in physical fitness values will be evaluated by analysis of variance (ANOVA). Training adherence data will be expressed as percentage. For all statistical comparisons, the significance level will be set at *P* < .05. Analyses of all metric data (ANOVA and paired *t* test) will be processed in the statistical software Statistica 12.

### 3.5 Trial status

This study respects World Medical Association Declaration of Helsinki on ethic in medical research and received approval by Ethical Committee of the UH Brno, Czech Republic. This trial inclines to SPIRIT 2013 checklist standards of reporting trials and is registered at Australian New Zealand Clinical Trial Registry with registration number: ACTRN12618001170213.

## 4 Discussion

Despite the fact that CR brings great benefits as a prevention of cardiovascular diseases,^[26,27]^ the regular outpatient training provided by health insurances is insufficient and physical fitness of patients usually gets worse after being discharged from the hospital.^[28]^ There are a lot of reasons why these patients refuse to train on their own. Besides the financial point of view (high expenses on individual coach), there are also problems in motivation, technical, and social issues.^[29]^ The average age of patients diagnosed with CAD is 60.4 ± 10.9 years.^[24]^ These people often suffer from depression of low mood coming from their disease and are afraid to manage their physical exercises themselves.^[13]^

Fast developing technology allows us to search for new possibilities. What we need to do is to find out how to use them in modern medicine. It is the telemedicine which has already been practiced and explored in many countries. For the Czech Republic, this way of remotely controlled patient is still unexplored and unique.

Already published foreign studies show how useful, low-cost, and successful telemedicine could be,^[30,31]^ some of them talk about even better results reached by telemedicine than center-based trainings.^[32]^ To distinguish from previous studies, we decided to try a new, in our country available tool—a wrist sport tester. Data transport from patients to physiotherapist is provided via Internet connection.

The aim of this study is to monitor 2 groups of patients (ROT and ITG groups) who will undergo a 12-week training. The ROT group will be monitored under the direct supervision by a physiotherapist in a hospital; the ITG will be monitored and checked via wrist sport tester during their home-based exercise trainings. What we focus on in our study is to compare the improvement of their quality of life and physical fitness. We expect that the improvement of both variables after 12 weeks of training will be statistically significant in both ROT and ITG groups.

In this way, we want to show how efficient and successful telemedicine is and suggest its usage in other fields of modern medicine where we can observe not only short-term but also mainly long-term and sustainable effects. Results of this study will be useful not only for UH Brno, Czech Republic, but also for other medical centers which have not been working with telemedicine yet. As the topic of telemedicine is quite new and unexplored in the Czech Republic, we would like to open a discussion about future possibilities. The results of our study could be the best way to begin.

## Author contributions

**Conceptualization:** Ladislav Batalik, Batalikova Katerina.

**Data curation:** Batalikova Katerina.

**Formal analysis:** Ladislav Batalik, Dosbaba Filip.

**Investigation:** Ladislav Batalik, Hartman Martin.

**Methodology:** Ladislav Batalik, Dosbaba Filip, Hartman Martin.

**Project administration:** Dosbaba Filip, Hartman Martin.

**Supervision:** Spinar Jindrich.

**Writing – original draft:** Ladislav Batalik.

**Writing – review and editing:** Batalikova Katerina.
